# Development of a quantitative fluorescence-based ligand-binding assay

**DOI:** 10.1038/srep25769

**Published:** 2016-05-10

**Authors:** Conor J. Breen, Mathilde Raverdeau, H. Paul Voorheis

**Affiliations:** 1 Department of Biology, Maynooth University, Maynooth, Co. Kildare, Ireland; 2 School of Biochemistry and Immunology, Trinity Biomedical Sciences Institute, Trinity College Dublin, Dublin 2, Ireland

## Abstract

A major goal of biology is to develop a quantitative ligand-binding assay that does not involve the use of radioactivity. Existing fluorescence-based assays have a serious drawback due to fluorescence quenching that accompanies the binding of fluorescently-labeled ligands to their receptors. This limitation of existing fluorescence-based assays prevents the number of cellular receptors under investigation from being accurately measured. We have developed a method where FITC-labeled proteins bound to a cell surface are proteolyzed extensively to eliminate fluorescence quenching and then the fluorescence of the resulting sample is compared to that of a known concentration of the proteolyzed FITC-protein employed. This step enables the number of cellular receptors to be measured quantitatively. We expect that this method will provide researchers with a viable alternative to the use of radioactivity in ligand binding assays.

Since the first radioligand binding assay was performed in 1970^1^, the technique has been widely used to characterize the number and specificity of cellular receptors. However, the desire to generate a cost-effective, risk-averse and environmentally friendly alternative to radioactivity has motivated researchers to develop a ligand-binding assay using fluorescently-labeled ligands rather than radiolabeled ligands^2^. Fluorophores such as fluorescein and its derivatives, particularly fluorescein 5’-isothiocyanate (FITC), are among those most commonly conjugated to proteins because they fulfil these criteria and have excellent absorption and emission properties^3^. Indeed, the dependence of fluorescence on temperature, pH and ionic strength can be overcome using thermostats and buffers, and by maintaining a constant ionic strength.

Nevertheless, existing fluorescence-based assays have a serious drawback due to the fluorescence quenching that accompanies the binding of fluorescently-labeled ligands to their receptors. This quenching is caused either by a conformational change in the fluorophore-conjugated ligand itself or a change in the oligomeric state of the ligand^4^,^5^. The fluorescein moieties of FITC-conjugated proteins are also substantially quenched by neighboring tryptophan, tyrosine, methionine and histidine residues within the same molecule^6^. The altered fluorescence emission arising from changes in inter- and intra-molecular quenching dramatically reduces the accuracy and sensitivity of any assay that relates the fluorescence intensity of FITC-conjugated proteins to their concentration. However, a possible way around these problems arose from the observation that separation and unfolding of fluorescein-labeled dimers with 8 M urea resulted in a ten-fold increase in fluorescence intensity^7^. Furthermore, proteolysis of intact fluorescein-labeled proteins also leads to the exposure of all of the conjugated fluorescein moieties and a resultant increase in fluorescence intensity^8^. These results raised the possibility that a quantitative measure of the concentration of an FITC-conjugated protein could be made from the fluorescence intensity if the quenched fluorescence of the FITC-conjugated protein in a cellular environment was completely “unquenched” by proteolysis prior to measurement.

The aim of this study was to develop a quantitative, fluorescence-based ligand-binding assay that avoids the following problems that are encountered with existing fluorescence-based binding assays: (1) an underestimate of the fluorescent ligand concentration due to intermolecular fluorescence quenching; (2) a reduced assay sensitivity due to an environment that reduces fluorescence, but promotes binding of the fluorescent ligand to its receptor; (3) an inaccurate estimate of the concentration of fluorescent ligand due to partial proteolytic degradation and/or reduction of disulfide bonds within a cellular environment, that only partially unquenches the fluorescence of the fluorescent ligand; (4) a reduced sensitivity of the assay due to intramolecular quenching of the fluorescent dye within the conjugated protein by neighboring amino acid residues.

We developed a “proteolytically-unquenched fluorescence” (PrUF) assay where both the bound FITC-conjugated protein under investigation and a known concentration of the same free FITC-conjugated protein standard are treated with pronase: a mixture of endo- and exo-peptidases with broad-range-specificity^9^,^10^ (Fig. 1). Under these conditions, we demonstrate that the proteolyzed FITC-conjugated proteins display identical fluorescence emission properties to an equimolar amount of FITC conjugated to the simple amine, methylamine (Supplementary Fig. S1). The amount of bound FITC-conjugated protein can therefore be calculated by comparing the fluorescence of the proteolyzed sample (containing the bound FITC-conjugated protein) to the fluorescence of a known concentration of the free proteolyzed FITC-conjugated protein. We anticipate that this method will provide researchers with a quantitative, straightforward and convenient alternative to radioligand-binding assays.

## Results

### The fluorescence of FITC-conjugated BSA, following proteolysis by the PrUF method, displays an identical dependence on the concentration of H^+^ to that of FITC-conjugated methylamine

**Figure 2:**
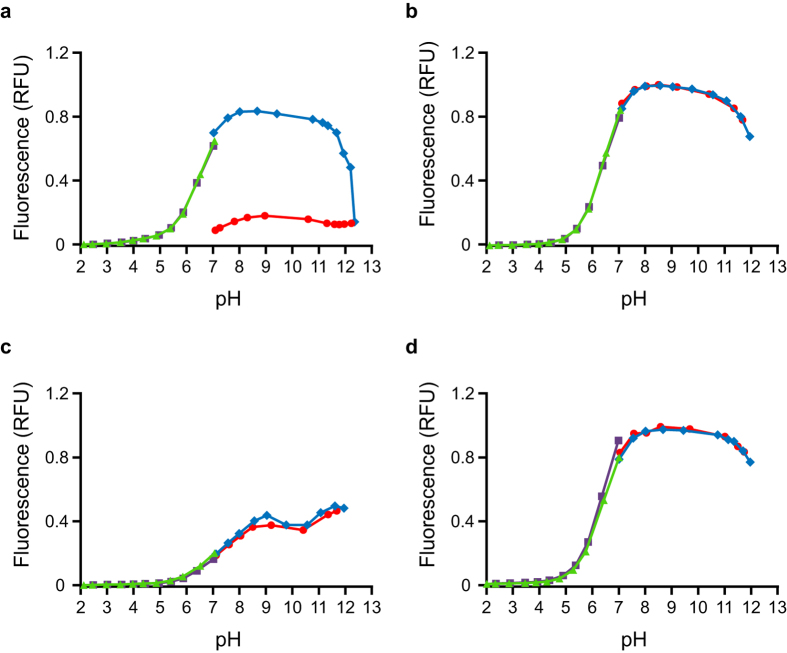
Proteolyzed FITC-BSA exhibits similar fluorescence to FITC-methylamine over a broad pH range. Fluorescence intensities of FITC **(a)**, FITC-methylamine **(b)**, FITC-BSA **(c)**, and proteolyzed FITC-BSA **(d)** as a function of pH: upward titrations from pH 7.0 with NaOH (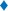) and return to neutral pH with HCl (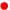), downward titrations from pH 7.0 with HCl (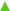) and return to neutral pH with NaOH (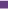). The fluorescein moiety of FITC itself irreversibly loses fluorescence at elevated pH (**a**). FITC-methylamine exhibits reversible loss of fluorescence at high and low pH (**b**) and has a fluorescence maximum at pH 8.5. The fluorescence of FITC-BSA (**c**) in response to changes in the H^+^ concentration is unlike that of FITC-methylamine (**b**) and has two fluorescence maxima, one at pH 8.5 and a second at pH 11.5. The fluorescence of proteolyzed FITC-BSA (**d**) in response to changes in the H^+^ concentration is similar to that of FITC-methylamine (**b**).

The response of the fluorescence intensity of FITC to changes in the H^+^ concentration clearly demonstrates that FITC irreversibly loses fluorescence at high pH (Fig. 2a). The irreversible loss of fluorescence of FITC at high pH is caused by the reaction of FITC with OH^−^ ions to form aminofluorescein and should be avoided^11^. However, when FITC is conjugated to a simple amine such as methylamine (Supplementary Fig. S1) the irreversible loss of fluorescence of the conjugated fluorescein moiety at high pH no longer occurs (Fig. 2b) since the thiourea linkage formed by the reaction of FITC with methylamine is not vulnerable to attack by OH^−^ ions. When FITC is conjugated to a more complex amine such as BSA (Uniprot: P02769), the response of the fluorescence intensity of FITC-BSA to changes in the H^+^ concentration (Fig. 2c) is markedly different to that of FITC-methylamine (Fig. 2b). However, when FITC-BSA is subjected to extensive proteolysis using pronase, the titration curves of proteolyzed FITC-BSA (Fig. 2d) and FITC-methylamine (Fig. 2b) are identical. This observation demonstrates that the unpredictable dependence of the fluorescence intensity of FITC-conjugated proteins on the H^+^ concentration can be overcome simply by treating the FITC-conjugated protein with pronase.

### Equimolar concentrations of the fluorescein tag in proteolyzed FITC-BSA and in FITC-methylamine possess identical fluorescence intensities

Next, we investigated the dependence of the fluorescence intensity of fluorescein on the complexity of the amine to which it is conjugated (Table 1). Interestingly, the fluorescence intensity of FITC itself was 22% lower than the fluorescence intensity of an equimolar amount of FITC-conjugated methylamine, presumably due to the reaction of free FITC with OH^−^ ions to form aminofluorescein in the time taken to perform the measurement^11^. The fluorescence intensity of native FITC-BSA was dramatically quenched compared to the fluorescence intensity of equimolar amounts of FITC-methylamine (6-fold lower). This quenching of the fluorescence intensity of FITC-BSA completely disappeared after treatment with pronase (Table 1). No further increase in the fluorescence intensity of pronase-treated FITC-BSA was detected upon addition of carboxypeptidase Y: a protease that cleaves proteins to the dipeptide stage^10^ (Table 1). Likewise, no further increase in the fluorescence of pronase-treated FITC-BSA was detected upon addition of dithiothreitol (DTT), which reduces disulfide bonds (Table 1).

### The proteolysis of FITC-BSA is unaffected by the presence of cells when sufficient pronase is employed

We investigated whether the proteolysis of a range of concentrations of FITC-BSA by pronase is affected by the addition of 10^8^ trypanosomes, which corresponds to 0.56 mg of cell protein^12^. The results demonstrated that there was no significant difference (P = 0.24) between the regression lines fitted to the fluorescence intensities of FITC-BSA that was proteolyzed in the presence and absence of 10^8^ trypanosomes (Supplementary Fig. S2)^13^. Clearly, the additional protein contributed by the cells themselves does not reduce the cleavage of bound FITC-conjugated protein ligand under the conditions of this assay because sufficient pronase has been employed.

### The fluorescence of fluorescein is stable when conjugated to an amine

FITC-methylamine did not lose its fluorescence at high pH over the short period required for the titration (Fig. 2b). In addition, the fluorescence intensity of FITC-methylamine at pH 8.5 only decreased by 0.39% over a period of 37 days (Supplementary Fig. S3). Assuming a linear decay, the t_1/2_ for the decay of fluorescence of FITC-conjugated methylamine was found to be 1286 days, or just over 3.5 years. However, if the decay of fluorescence follows a first-order mechanism then the t_1/2_ would be even longer.

Clearly, to determine the t_1/2_ of the decay of fluorescence of FITC-methylamine accurately, the fluorescence would have to be measured over a much greater time period. However, we can conclude that during the period of time between the synthesis, purification and the subsequent use of FITC-conjugated amines in an assay the decay of the fluorescence of the conjugate would be negligible even if samples were stored for a year before measurement.

### Bloodstream forms of *Trypanosoma brucei* contain high-capacity, medium-affinity binding sites for FITC-conjugated holotransferrin and apotransferrin, exclusively in the flagellar pocket

Previous reports have demonstrated that iron-rich holotransferrin (Uniprot: Q29443) is a growth factor for bloodstream forms of *T. brucei*^14^,^15^,^16^, the parasite responsible for African sleeping sickness. Studies have also indicated that iron-poor apotransferrin can bind to bloodstream forms of *T. brucei* but that this binding is inhibited at low pH^16^,^17^. Radioligand binding assays have been used to estimate the number of specific holotransferrin binding sites per cell but these estimates vary by almost two orders of magnitude between different laboratories^14^,^15^,^16^. By contrast, no studies have measured the number of apotransferrin binding sites per cell. Therefore, we carried out quadruplicate independent saturation binding experiments using the novel PrUF method described in this report to measure the number and affinity of bovine holotransferrin and apotransferrin binding sites present on the surface of bloodstream forms of *T. brucei*.

Equilibrium binding conditions were established by conducting a time course of binding of both FITC-holotransferrin and FITC-apotransferrin to the cells (Supplementary Figs S4a and b). The time taken for equilibrium to be reached (defined here as the time taken to reach 95% occupancy) was t_95%_ = 4.3 min for FITC-holotransferrin and t_95%_ = 7.2 min for FITC-apotransferrin (n = 3). However, the actual time taken to reach equilibrium is likely to be even shorter since maximum binding was observed as soon as the first measurement could be performed due to the inherent limitations of the centrifugation step within the assay. Nevertheless, these estimates allow us to establish with confidence that equilibrium has been reached within this period of time.

**Figure 3:**
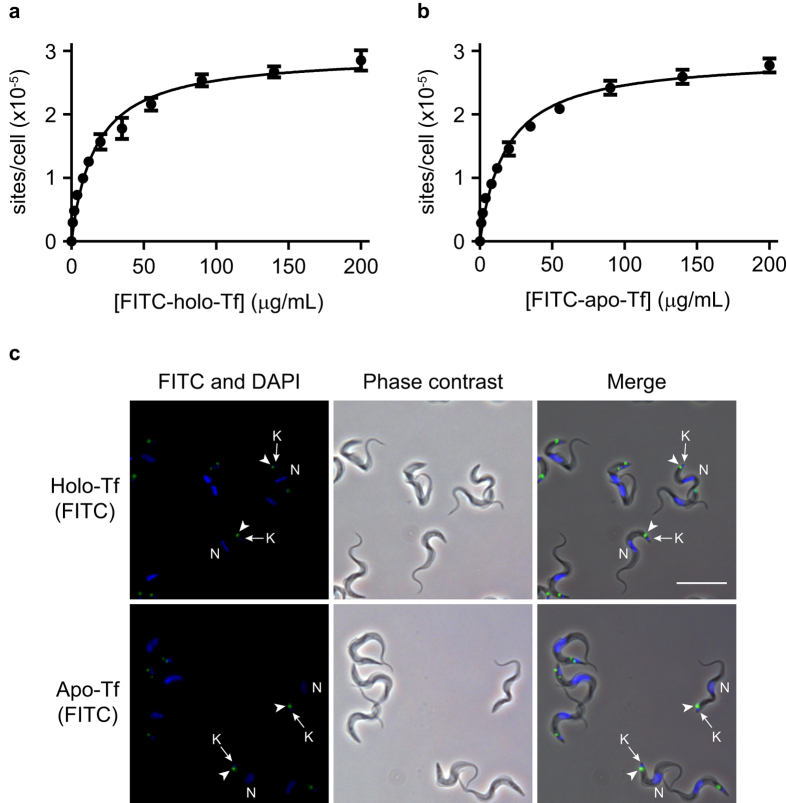
Binding of both FITC-holotransferrin and FITC-apotransferrin to bloodstream form trypanosomes is saturable and localized to the flagellar pocket. A one-site binding model (equation 1) was fitted to the data for the binding isotherms of FITC-holotransferrin (**a**) and FITC-apotransferrin (**b**). Data are the mean ± S.D. of four independent experiments. Fluorescence and phase contrast micrographs show that FITC-holotransferrin and FITC-apotransferrin bound exclusively to the flagellar pocket region of bloodstream form trypanosomes (**c**). The location of apotransferrin and holotransferrin staining (green) is indicated by arrowheads. The positions of the nuclei (N) and kinetoplasts (K) are indicated by arrows and were visualized by staining with Hoechst 33342 (blue). Scale bar, 20 μm.

One-site binding models were fitted to the equilibrium binding isotherms for FITC-holotransferrin (Fig. 3a) and FITC-apotransferrin (Fig. 3b) on bloodstream form trypanosomes. These models predicted high-capacity, medium affinity binding sites for both holotransferrin [B_max_ = (2.96 ± 0.06) × 10^5^ sites/cell; K_D_ = 190.5 ± 2.4 nM] and apotransferrin [B_max_ = (2.91 ± 0.05) × 10^5^ sites/cell; K_D_ = 209.4 ± 2.1 nM] (mean ± s.d.; n = 4). We did not detect any difference between the number of binding sites for holotransferrin and apotransferrin on bloodstream form trypanosomes (Student’s unpaired two-tailed t-test; n = 4, P = 0.25). However, a minor but significant increase (9%) was observed in the affinity of holotransferrin for cells compared to apotransferrin (Student’s unpaired two-tailed t-test; n = 4, P < 0.0001).

Receptor-mediated endocytosis in *T. brucei* takes place exclusively from the flagellar pocket: an invagination of the plasma membrane from which the flagellum emerges^14^. This structure is posterior to the nucleus and immediately above the kinetoplast: the site where mitochondrial DNA is stored. Since proteins are internalized only from the flagellar pocket, the location of FITC-holotransferrin and FITC-apotransferrin binding sites on the surface of bloodstream form trypanosomes was determined using fluorescence microscopy (Fig. 3c) as an additional control for the validity of our observations. The fluorescent micrographs confirmed that both holotransferrin and apotransferrin bind to a localized spot in the region of the kinetoplast, confirming previous reports that holotransferrin and apotransferrin bind exclusively to the flagellar pocket^16^,^18^.

## Discussion

Ligand-binding assays are fundamental to our understanding of the properties of receptors but existing fluorescence-based binding assays suffer from intra- and intermolecular quenching of the fluorescent ligand. Our results demonstrate that the concentration of FITC-conjugated protein bound to a cell sample can be determined after removing the unbound probe by treating the washed cells with pronase and comparing the fluorescence of the proteolyzed sample to the fluorescence of a known concentration of the free proteolyzed FITC-conjugated protein. The PrUF methodology can also be used to determine the fluorophore:protein labeling ratio (F:P ratio) of FITC-conjugated proteins by comparing the fluorescence of the proteolyzed FITC-conjugated protein to that of a known concentration of FITC-methylamine in conjunction with a protein assay such as the BCA or Lowry assay. We have found that the traditional use of absorbance measurements to measure F:P ratios^19^ leads to underestimation of the degree of labeling when high concentrations of the fluorophore are used in the labeling reaction which is probably due to increased intramolecular quenching that occurs at high F:P ratios.

Although FITC-conjugated proteins have been used throughout this study, we expect that other fluorophores could also be used as long as total unquenching of the fluorescently-labeled protein can be demonstrated under the conditions that the assay will be performed. Similarly, the PrUF methodology should also be applicable to other cell and tissue types as long as complete proteolysis can be achieved in the system under investigation. In this study, complete unquenching of FITC-conjugated proteins has been achieved using pronase: a mixture of endo- and exo-peptidases that cleaves proteins down to single amino acids or small peptides. However, other proteases and proteolysis buffers could be explored when attempting to adapt the PrUF method to other systems. For instance, proteinase K has the advantage that it hydrolyzes a variety of peptide bonds over a wide pH and temperature range and in the presence of denaturing agents such as SDS and urea, which may facilitate unquenching. Optimizing the proteolysis conditions is therefore essential to ensure rapid and complete unquenching of fluorescently-labeled proteins when bound to other cell and tissue types.

In this study we also provide an explanation for the unresolved conflict between previous reports that obtained strikingly different estimates of the number of holotransferrin binding sites per trypanosome, despite agreement that FITC-conjugated holotransferrin binds to bloodstream forms of *T. brucei* with saturation kinetics^14^,^15^ (Fig. 3a). Coppens *et al*. obtained a value of 1.5 × 10^5^ binding sites per cell for ^125^I-bovine holotransferrin in the absence of competing heterologous ligand^14^, which is half of the total number of holotransferrin binding sites (2.96 × 10^5^ per cell) estimated in this study. One possible explanation for the lower value obtained in the above study might be due to the loss of cells during the centrifugation stage. We observed that the cell pellet frequently extended up the side of the tube, leading to a loss of cells during aspiration of the supernatant. However, rotation of the tube, followed by a short re-centrifugation step resulted in a compact, stable pellet. In another study, Salmon *et al*. reported a range of 1.9 × 10^4^ and 2.7 × 10^4^ holotransferrin binding sites per cell in the presence of an excess of unlabeled ovalbumin to prevent non-specific binding^15^. An even lower estimate of 2.3 × 10^3^ holotransferrin binding sites per cell was obtained by Steverding *et al*. when the binding of ^3^H-bovine holotransferrin was analysed in the presence of an excess of fish gelatin^16^. The present study has demonstrated by immunofluorescence microscopy that FITC-conjugated holotransferrin binds exclusively to the region between the nucleus and the kinetoplast, even in the absence of competing unlabeled ligand. Furthermore, when the competitive heterologous ligand is an unlabeled protein and its concentration is varied over a wide range, we have found it to display a strictly competitive inhibition pattern with respect to the full concentration range of the FITC-conjugated ligand, suggesting it binds to all binding sites equally. These two findings argue strongly against the classification of the high-capacity, medium-affinity holotransferrin and apotransferrin binding sites as non-specific binding as opposed to a separate set of specific transferrin binding sites.

In conclusion, we have described a novel, unquenched, fluorescence methodology that is unaffected by changes in the fluorescence intensity of the FITC-conjugated ligand that usually accompanies binding of the ligand to its receptor. We envisage that this new PrUF assay can be adapted to a wide range of cell and tissue types and will provide researchers with a viable alternative to the use of radioactivity.

## Methods

### Reagents and materials

All reagents and buffers were from Sigma-Aldrich. Polymethylacrylate cuvettes (3 mL capacity) were from Sarstedt. OptiPlate 96-F fluorescence microplates (black) were from Perkin Elmer.

### FITC labeling of methylamine and proteins

Methylamine, holotransferrin, apotransferrin and BSA were dissolved in sodium carbonate, (0.1 M; pH 9.1) and transferred to a small vessel. FITC was dissolved in DMSO, diluted with carbonate buffer and added drop-wise to the solution of protein. Methylamine was labeled using a 50-fold molar excess of methylamine relative to the concentration of FITC, while holotransferrin, apotransferrin and BSA were labeled using a 100-fold molar excess of FITC. The labeling reaction was allowed to proceed for 3 h in the dark at room temperature with constant gentle stirring. Unreacted FITC was separated from the FITC-conjugated proteins (prepared using a 100-fold molar excess of FITC) by passage through a column of Sephadex G-25 (0.8 cm × 42 cm; medium grade; Sigma) that had previously been equilibrated with TES buffer (30 mM TES, 140 mM NaCl, 4 mM KCl, pH 7.4). A second batch of FITC-BSA was prepared using a 7.5-fold molar excess of FITC relative to the concentration of BSA. Since there are 60 lysine residues in BSA, this batch contained an 8-fold molar excess of lysine residues compared to the concentration of FITC and was used to compare the fluorescence of equimolar amounts of the fluorescein tag under different conditions (Fig. 2a–d and Table 1).

### Fluorescence spectroscopy

Fluorescence emission was monitored in cuvette format, using an LS-50B fluorescence spectrometer (Perkin Elmer) or in a microplate format, using a POLARstar Omega microplate reader (BMG Labtech). For measurements made using the LS-50B, slit widths on the excitation and emission monochromators were set to 5 nm and an integration time of 0.2 s was chosen. Polymethylacrylate cuvettes, with a 3 mL capacity, were used throughout the study. Constant temperature was maintained by a circulating water bath connected to the cuvette holder. The excitation and emission wavelengths used for FITC and FITC-conjugated amines were determined from spectral scans (see Supplementary Methods and Supplementary Fig. S5a–d). Measurements with the POLARstar Omega were made using an excitation filter of 485 nm and an emission filter of 520 nm. The samples were measured using the top optic with 10 flashes per well and a gain set to 1000. OptiPlate 96-F fluorescence microplates (black) were used throughout.

### pH titration of FITC and FITC-conjugated amines

FITC, FITC-methylamine, FITC-BSA (prepared using an excess of amine) and FITC-BSA (after proteolysis) were diluted in a broad-range buffer (10 mM BES, 10 mM Bicine, 10 mM formic acid, 10 mM malic acid, 10 mM MES; pH 7) before use. The samples were then titrated in the upward direction with NaOH (0.5 M) and then back to neutral pH with HCl (0.5 M). A second set of samples were titrated in the downward direction with HCl (0.5 M) and then back to neutral pH with NaOH (0.5 M). After each addition of HCl or NaOH, the contents of the cuvette were mixed thoroughly. The volume of acid or base added to the cuvette was varied (5 to 50 μL) so that the measurements were distributed evenly across the entire range of pH. The fluorescence was monitored after each addition of acid or base and the fluorescence was corrected for the consequent change in volume. The pH of the solution after the addition of acid or base was determined in a parallel experiment because FITC conjugates were found to adsorb to the pH electrode. Fluorescence intensities were measured as described previously.

### Determining the fluorescence of FITC and FITC-conjugated amines

FITC, FITC-methylamine and FITC-BSA (prepared using an excess of amine as described previously) were diluted in proteolysis buffer (30 mM TES, 140 mM NaCl, 4 mM KCl, 10 mM CaCl_2_; pH 7.5) and supplemented with pronase (100 μg/mg of FITC-conjugated protein. These samples were incubated in the dark for 72 h at 37 °C. Some of the pronase-treated FITC-conjugated BSA was then treated with 0.5 mg carboxypeptidase Y for 8 h at 30 °C or with a 100-fold molar excess of dithiothreitol relative to the concentration of protein. All samples were then diluted to the same concentration of the fluorescein label (257 nM) in Bicine-NaCl buffer (100 mM Bicine, 100 mM NaCl; pH 8.5) and their fluorescence intensities measured as described previously.

### Determination of protein concentration

Protein concentrations were determined using the Lowry method^20^. Known concentrations of BSA, holotransferrin and apotransferrin were used as protein standards as appropriate.

### Determination of fluorescein/protein molar ratios

FITC-methylamine was prepared with a 100-fold molar excess of methylamine relative to the concentration of fluorescein, using the methodology described above. The fluorescence of this preparation was determined using a spectrofluorimeter and compared to the fluorescence of pronase-treated FITC-holotransferrin and FITC-apotransferrin (100 μg pronase/10 μg FITC-protein). The ratio of the fluorescence of equimolar amounts of FITC-labeled protein and that of FITC-labeled methylamine was used to determine the fluorescein:protein molar ratio of the protein ligands. Using this methodology the fluor:protein molar ratios of FITC-holotransferrin and FITC-apotransferrin were 10.8 ± 0.26 and 11.6 ± 0.45 (mean ± s.d.; n = 4), respectively.

### Source and purification of trypanosomes

Growth, isolation and measurement of the concentration of trypanosomes has been described previously^12^.

### Fluorescence-based ligand-binding assay

Following purification of bloodstream form trypanosomes by DEAE chromatography, the cells were centrifuged in a swing-out rotor at 1200 RPM for 10 min at 4 °C and the pellet of cells resuspended in ice-cold TES cell buffer (30 mM TES, 120 mM NaCl, 5 mM KCl, 16 mM Na_2_HPO_4_, 1.2 mM KH_2_PO_4_, 3 mM MgSO_4_, 10 mM glucose, 0.1 mM adenosine; pH 7.4). The cells were then counted at least three times in a haemocytometer and the volume adjusted to give 2 × 10^8^ cells/mL. Samples (500 μL) containing 10^8^ trypanosomes were then transferred to equal volumes of either FITC-conjugated holotransferrin or FITC-conjugated apotransferrin (0–200 μg). Following incubation for 20 min on ice, the cells were centrifuged at 12,000 × *g* for 30 s at 4 °C. The tubes containing the cells were then rotated by 180° and re-centrifuged to ensure a tight pellet at the bottom of the tube. The supernatants were then aspirated to waste and the pellets resuspended in ice-cold TES cell buffer (1 mL). The trypanosomes were washed once more in this manner, and following a third centrifugation step were finally resuspended in a calcium-containing, phosphate-free TES proteolysis buffer (30 mM TES, 140 mM NaCl, 4 mM KCl, 10 mM CaCl_2_; pH 7.5) containing pronase (100 μg pronase/490 μL buffer). These samples were incubated in the dark for 72 h at 37 °C to completely unquench the fluorescence of the bound FITC-conjugated proteins (Supplementary Fig. S6a and b). In addition, a known concentration of the FITC-conjugated protein under investigation was proteolyzed in parallel using the same conditions. The proteolyzed samples were then diluted in Bicine-NaCl buffer (100 mM Bicine, 100 mM NaCl; pH 8.5) and their fluorescence intensities measured using the POLARstar Omega microplate reader as previously described. The amount of bound FITC-conjugated protein was obtained by comparing the fluorescence intensities of the samples in the binding experiment to the fluorescence of a known amount of the free proteolyzed FITC-conjugated protein.

### One-site ligand binding

The exact mathematical expression used to estimate the concentration of ligand to a single binding site is given by the solution of the following quadratic equation and taking the negative root:


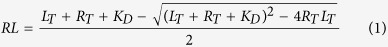


where *L*_*T*_ and *R*_*T*_ describe the total ligand and receptor concentration, respectively, and *K*_*D*_ describes the dissociation constant. This model is described in detail by Stein *et al*.^21^. The axes labels of the relevant figures were then converted from μM to μg/mL (x-axis) and from pmoles to sites/cell (y-axis).

### Fluorescence and phase contrast microscopy

A sample of 10^8^ freshly isolated bloodstream form trypanosomes was incubated with FITC-holotransferrin or FITC-apotransferrin (100 μg) in ice-cold TES buffer (30 mM TES, 120 mM NaCl, 5 mM KCl, 16 mM Na_2_HPO_4_, 1.2 mM KH_2_PO_4_, 3 mM MgSO_4_, 10 mM glucose, 0.1 mM adenosine; pH 7.4) for 20 min. The cells were then centrifuged at 12,000 × *g* for 30 s, the supernatants aspirated to waste and the pellets resuspended in ice-cold glucose-/adenosine-free TES buffer (1 mL). The cells were washed once more in this manner and after a third centrifugation step were resuspended in ice-cold TES buffer (500 μL). An equal volume of ice-cold paraformaldehyde (6% w/v) was added to the cells for 15 min at 0 °C. The cells were then centrifuged at 12,000 × *g* for 30 s and the supernatants aspirated to waste. The trypanosomes were resuspended at a final concentration of 2 × 10^7^ cells/mL in ice-cold, glucose-/adenosine-free TES buffer containing sodium azide (15 mM) before incubation on poly-L-lysine-coated coverslips (100 μL per coverslip) for 15 min at room temperature. The coverslips were then washed with an anti-photobleaching solution (1 mg/mL p-phenylenediamine, 50% (v/v) PBS, 50% (v/v) glycerol) and after the majority of the excess fluid removed with a pipette; the coverslips were mounted onto slides and sealed with nail varnish.

Slides were examined by fluorescence and phase contrast microscopy with an upright Axiovert 100TV Zeiss Microscope. The excitation light for imaging was provided by a mercury lamp through a 40x oil objective and captured at a resolution of 1300 × 1030 pixels (5.9 pixels/μm; 8 bits/pixel in *.tif format) by a cooled CCD camera (Axiocam MR; Zeiss) using the Axiovision software (Version 1.3c). Images were cropped to (472 × 472 pixels) and the DAPI and FITC channels merged using the “merge channels” function in ImageJ (v1.46r). The fluorescence and phase contrast images were merged in Adobe Photoshop CS6 by superimposing the phase contrast image over the fluorescent image and reducing the opacity of the phase contrast layer to 20%. The brightness of the merged image was then increased uniformly by a factor of 3.375 (brightness increased from 0 to 150).

## Additional Information

**How to cite this article**: Breen, C. J. *et al*. Development of a quantitative fluorescence-based ligand-binding assay. *Sci. Rep*. **6**, 25769; doi: 10.1038/srep25769 (2016).

## Supplementary Material

Supplementary Information

## Figures and Tables

**Figure 1:**
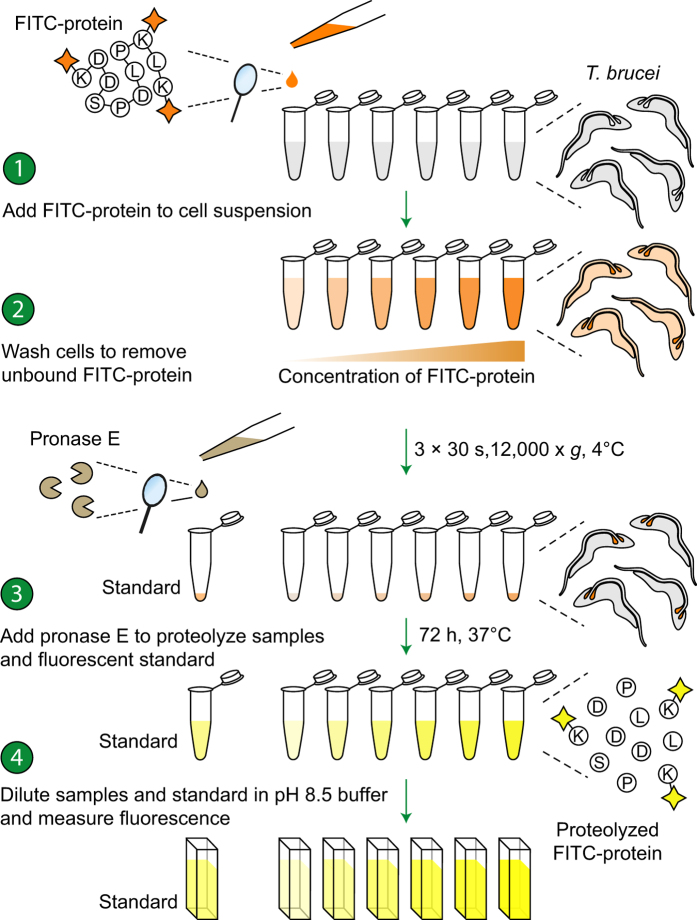
Schematic step diagram describing the PrUF-based ligand-binding assay. Cells were incubated with varying concentrations of the FITC-conjugated protein of interest in isosmotic TES buffer, washed free from unbound protein and diluted into Ca^2+^-containing proteolysis buffer with pronase. The samples were then diluted into Bicine buffer and the fluorescence measured in a spectrofluorimeter. The concentration of FITC-conjugated protein in each sample was determined by comparing its fluorescence to that of a known concentration of the proteolyzed FITC-conjugated protein standard.

**Table 1:** Equimolar concentrations of the fluorescein moiety in proteolyzed FITC-BSA and pronase-treated FITC-methylamine have the same fluorescence intensity.

Equimolar amounts of conjugated and unconjugated fluorescein tag	λ_exc_	λ_em_	Fold change compared to fluorescence of FITC-methylamine	P values(n = 3)
FITC-methylamine	490 nm	514 nm	1.00 ± 0.02	—
FITC-methylamine treated with pronase E	490 nm	514 nm	0.99 ± 0.06	0.88
FITC-BSA	490 nm	520 nm	0.17 ± 0.01	2.69 × 10^−4^
FITC-BSA treated with pronase E	490 nm	514 nm	1.01 ± 0.04	0.83
FITC-BSA treated with pronase E and carboxypeptidase Y	490 nm	514 nm	1.00 ± 0.02	0.53
FITC-BSA treated with pronase E and DTT	490 nm	514 nm	1.02 ± 0.05	0.53
FITC	490 nm	517 nm	0.83 ± 0.01	2.90 * 10^−3^
Aminofluorescein	487 nm	507 nm	6.80 * 10^−3^ ± 1.13 * 10^−4^	5.78 * 10^−5^

Data are the mean ± S.D. (n = 3). The fold-change represents the change in fluorescence intensity of the treatment compared to the fluorescence of FITC-methylamine without treatment. Excitation and emission wavelengths were obtained as described in Supplementary methods.
