# Supplementary material for: Development of a quantitative fluorescence-based ligand-binding assay
Source: Sci Rep. 2016 May 10;6:25769. doi: 10.1038/srep25769 (PMC4861924; doi:10.1038/srep25769)
Supplement: Supplementary Information [file srep25769-s1.pdf]

Development of a quantitative fluorescence-based ligand-binding assay

Conor J. Breen[1, 2, *], Mathilde Raverdeau[2] & H. Paul Voorheis[2]

[1] Department of Biology, Maynooth University, Maynooth, Co. Kildare, Ireland

[2] School of Biochemistry and Immunology, Trinity Biomedical Sciences Institute, Trinity College Dublin, Dublin 2, Ireland

*Correspondence should be addressed to Conor J. Breen (Conor.Breen@nuim.ie)

Supplementary figures

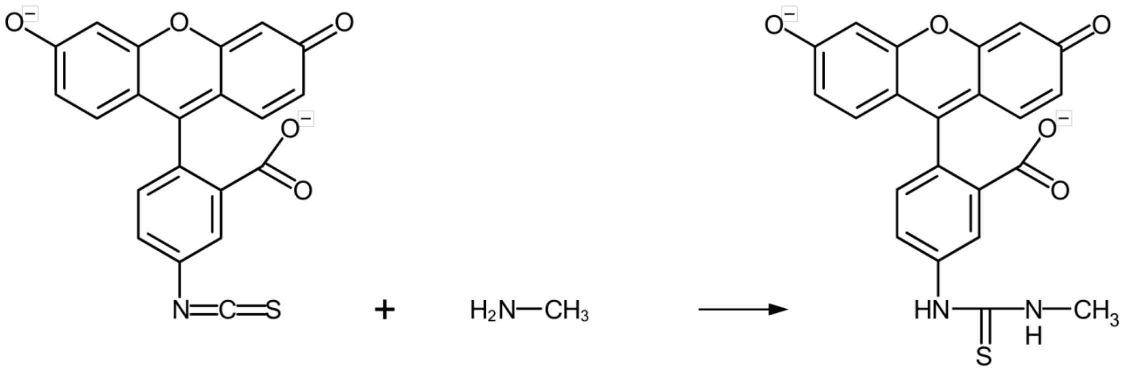

FITC methylamine FITC-methylamine

Supplementary Figure S1: The reaction between FITC and methylamine

The reaction between the isothiocyanate group of FITC and the primary amine of methylamine is shown in the figure. FITC is most commonly used to label proteins, where it reacts with the primary amines that are found both on both the side chains of lysine residues and at the N-terminus of the protein.

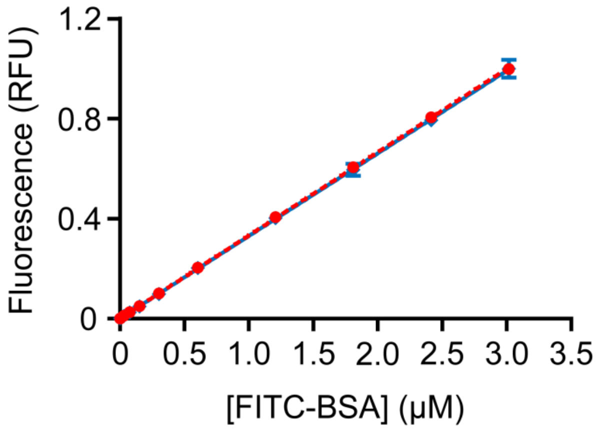

Supplementary Figure S2: Proteolysis of FITC-BSA by an excess of pronase E is unaffected by the presence of 10^8 trypanosomes.

The calculated regression lines are shown through the fluorescence intensity data of various concentrations of FITC-BSA that had been previously treated with pronase in the absence (♦) and presence (●) of 10^8 trypanosomes. Data are the mean ± S.D. of the fluorescence intensity (n=3).

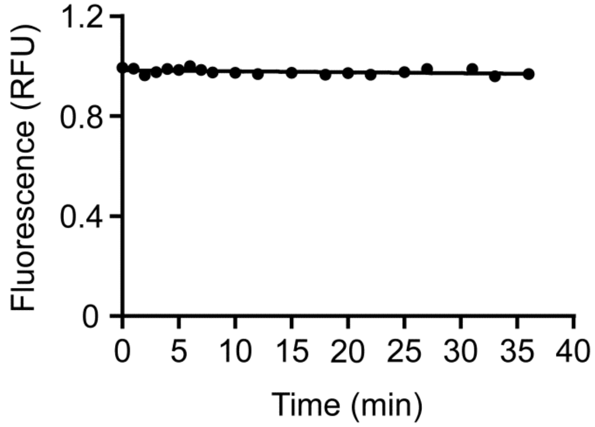

Supplementary Figure S3: Fluorescence of FITC-methylamine is largely unaffected by prolonged storage.

Samples were taken regularly from a solution of FITC-methylamine, stored in the dark,

and their fluorescence intensities measured and plotted as a function of time. Data are the mean ± S.D. of the fluorescence intensity (n=3).

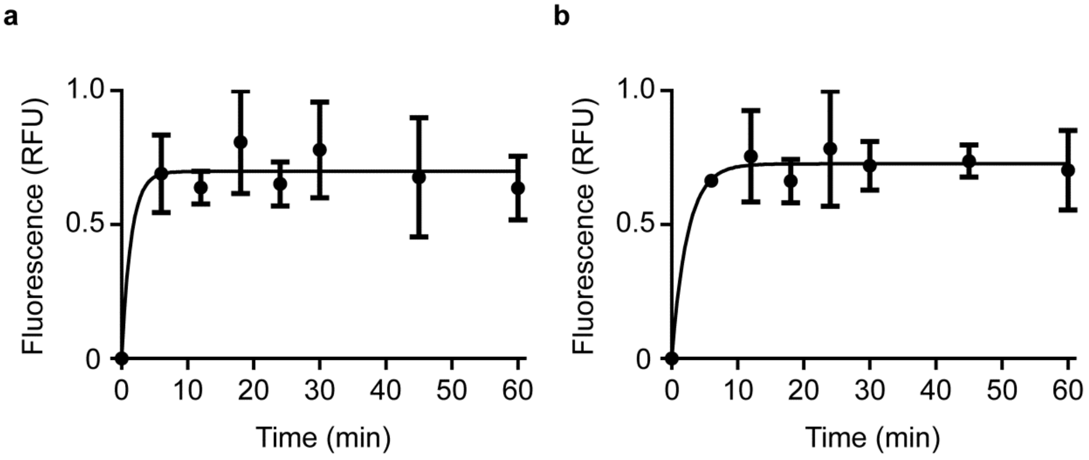

Supplementary Figure S4: Time courses of binding of FITC-holotransferrin (a) and FITC-apotransferrin (b) to bloodstream form trypanosomes.

Pseudo-first order kinetic models (equation 2) were fitted to the data (solid line) as indicated in Supplementary Methods. The time taken to reach 95% occupancy was calculated by solving the integrated form of the pseudo-first order rate equation (equation 3).

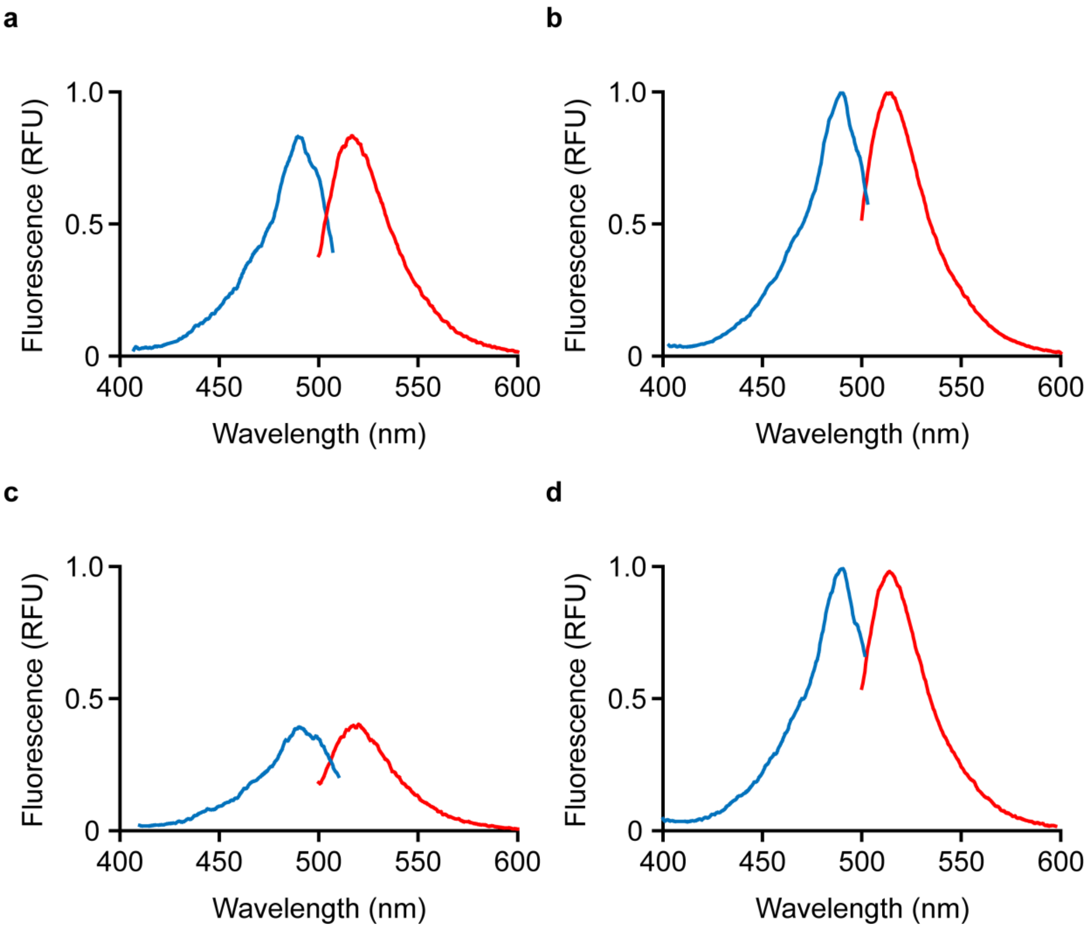

Supplementary Figure S5: Excitation (blue trace) and emission (red trace) spectra of FITC (a), FITC-methylamine (b), FITC-BSA (c), and proteolyzed FITC-BSA (d). Equal amounts of the fluorescein label were used between conditions. The wavelengths of maximum excitation (λ_{exc}) and emission (λ_{em}) are summarized in **Table 1**.

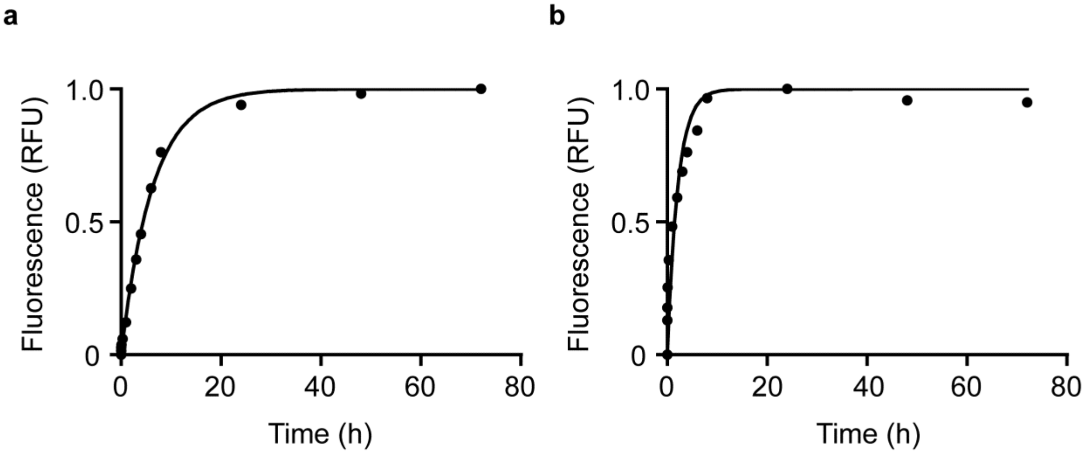

Supplementary Figure S6: Time courses of the pseudo-first order proteolysis of FITC-holotransferrin (a) and FITC-apotransferrin (b).

Samples of 40 µg of the FITC-conjugated protein were digested with 100 µg of pronase in the presence of 960 µg of unlabeled BSA. Pseudo-first order kinetic models were fitted to the data (solid line) as indicated in Supplementary Methods (equation 2). The time taken to reach 95% proteolysis was then calculated by solving the integrated form of the pseudo-first order rate equation (equation 3). Under the conditions described above, the time taken to reach 95% proteolysis was 18.9 h for holotransferrin and 6.4 h for apotransferrin.

Supplementary Methods

Time courses of binding of FITC-holotransferrin and FITC-apotransferrin to bloodstream form trypanosomes

Bloodstream form trypanosomes (10^8) were incubated with the FITC-conjugated protein (2 µg) in ice-cold TES buffer (30 mM TES, 120 mM NaCl, 5 mM KCl, 16 mM Na_2HPO_4, 1.2 mM KH_2PO_4, 3 mM $MgSO_4$, 10 mM glucose, 0.1 mM adenosine; pH 7.4)

for various times. After incubation, the cells were centrifuged at 12,000 × g for 30 s and the supernatants aspirated to waste. The pellets of trypanosomes were resuspended in ice-cold TES buffer (1 mL) and washed once more in this manner. Following a third centrifugation step, the trypanosomes were finally resuspended in TES proteolysis buffer (30 mM TES, 140 mM NaCl, 4 mM KCl, 10 mM $CaCl_2$; pH 7.5) containing pronase (100 µg/490 µL) and then incubated in the dark for 72 h at 37°C. The proteolyzed samples were then diluted in Bicine-NaCl buffer (100 mM Bicine, 100 mM NaCl, pH 8.5) and their fluorescence intensities measured as previously described. Pseudo-first order reaction functions (equation 2) were fitted to the data from the time courses of binding:

$$y = A(1 - e^{-kt}) \tag{2}$$

where A is the plateau and k is the pseudo-first order rate constant. The times taken to reach 95% occupancy were then calculated by solving the integrated form of the rate equation (equation 3) for 95% of the maximum fluorescence intensities:

$$t_{95\%} = \frac{\ln(\frac{1}{0.05})}{k} \tag{3}$$

where k is the pseudo-first order rate constant.

Time course of proteolysis of FITC-conjugated proteins by pronase

FITC-holotransferrin and FITC-apotransferrin (100-fold initial molar excess of FITC) were prepared as described in Methods. Both FITC-conjugated proteins (40 µg) were

proteolyzed in the presence of 960 μg unlabeled BSA in proteolysis buffer (30 mM TES, 140 mM NaCl, 4 mM KCl, 10 mM $CaCl_2$; pH 7.5) containing pronase (100 μg), for 4 days in the dark at 37°C. The reaction was terminated at various times by dilution into Bicine-NaCl buffer (100 mM Bicine, 100 mM NaCl; pH 8.5) at 0°C containing BSA (2 mg/mL) and protease inhibitors (50 mM EDTA, 2 mM PMSF and 100 μg/mL leupeptin, pH 8.5) and their fluorescence intensities measured as previously described. Pseudo-first order reaction functions were fitted to the data from the time courses of proteolysis as described above (equation 2). The times taken for the reactions to reach 95% completion were then calculated by solving the integrated form of the rate equation for 95% of the maximum fluorescence intensities as described above (equation 3).

Excitation and emission spectra

The maximum excitation and emission wavelengths of FITC, FITC-methylamine, FITC-BSA, and proteolyzed FITC-BSA were determined by serial dilution of each component into Bicine-NaCl buffer (100 mM Bicine, 100 mM NaCl, pH 8.5) and measuring the fluorescence intensities as described in Methods. The scan speed was 100 nm/min and the excitation and emission slit widths were set to 5 nm. All measurements were made at 25°C.
